# A large-scale multiomics analysis of wheat stem solidness and the wheat stem sawfly feeding response, and syntenic associations in barley, *Brachypodium*, and rice

**DOI:** 10.1007/s10142-017-0585-5

**Published:** 2018-02-22

**Authors:** Sezgi Biyiklioglu, Burcu Alptekin, B. Ani Akpinar, Andrea C. Varella, Megan L. Hofland, David K. Weaver, Brian Bothner, Hikmet Budak

**Affiliations:** 1 Cereal Genomics Laboratory, Department of Plant Sciences and Plant Pathology, Montana State University, Bozeman, MT USA; 2 Department of Plant Sciences and Plant Pathology, Montana State University, Bozeman, MT USA; 3 Wheat Stem Sawfly Laboratory, Department of Land Resources and Environmental Sciences, Montana State University, Bozeman, MT USA; 4 Department of Chemistry and Biochemistry, Montana State University, Bozeman, MT USA

**Keywords:** *Brachypodium*, *Hordeum vulgare*, Insect resistance, *Oryza sativa*, Stem solidness, *SSt1*, *Triticum aestivum*, Wheat stem sawfly feeding

## Abstract

The wheat stem sawfly (WSS), *Cephus cinctus* Norton (Hymenoptera: Cephidae), is an important pest of wheat and other cereals, threatening the quality and quantity of grain production. WSS larvae feed and develop inside the stem where they are protected from the external environment; therefore, pest management strategies primarily rely on host plant resistance. A major locus on the long arm of wheat chromosome 3B underlies most of the variation in stem solidness; however, the impact of stem solidness on WSS feeding has not been completely characterized. Here, we used a multiomics approach to examine the response to WSS in both solid- and semi-solid-stemmed wheat varieties. The combined transcriptomic, proteomic, and metabolomic data revealed that two important molecular pathways, phenylpropanoid and phosphate pentose, are involved in plant defense against WSS. We also detected a general downregulation of several key defense transcripts, including those encoding secondary metabolites such as DIMBOA, tricetin, and lignin, which suggested that the WSS larva might interfere with plant defense. We comparatively analyzed the stem solidness genomic region known to be associated with WSS tolerance in wild emmer, durum, and bread wheats, and described syntenic regions in the close relatives barley, *Brachypodium*, and rice. Additionally, microRNAs identified from the same genomic region revealed potential regulatory pathways associated with the WSS response. We propose a model outlining the molecular responses of the WSS–wheat interactions. These findings provide insight into the link between stem solidness and WSS feeding at the molecular level.

**Electronic supplementary material:**

The online version of this article (10.1007/s10142-017-0585-5) contains supplementary material, which is available to authorized users.

## Introduction

Wheat (*Triticum aestivum*) is a staple food for 30% of the global population and is the most extensively grown crop in the world, ranking third in terms of production with an annual output of over 700 million tons (http://faostat3.fao.org/). Biotic stresses are responsible for up to 20% of the yield loss in wheat, presenting a major obstacle to achieving the goal of sustainably providing enough yield to feed the world. The wheat stem sawfly (WSS; *Cephus cinctus* Norton) is a major pest of winter and spring hexaploid wheat and tetraploid durum wheat (*T*. *turgidum* ssp. *durum*), which are harvested across the Northern Great Plains of North America (Lesieur et al. 2016).

WSS completes its life cycle within a year, most of which is spent in the larval stages. The adult first emerge from the previous year’s stub in early summer, typically in mid to late June (Beres et al. 2011b; Nilsen et al. 2016). Female adults, which live for 7–10 days, lay their eggs inside the wheat stem, and the resulting larvae begin feeding on the parenchyma and vascular tissues. Following the fifth instar stage, the larvae move down the inside of the stem and cut the stem at the base. The toppled stem provides shelter for the larvae, allowing them to overwinter during the obligatory diapause stage (Beres et al. 2011a). Heavy WSS infestation can cause up to 95% of the stems to lodge due to this stem-cutting behavior (Beres et al. 2011b). The combination of lodging and decreased photoassimilation due to vascular injury may account for yield losses as high as 30%, equating to economic losses of $350 million annually (Delaney et al. 2010; Beres et al. 2011a; Nilsen et al. 2016). Since the larvae are protected inside the stems and the adult flies do not feed, chemical control strategies against WSS are ineffective. Insecticides can instead affect the natural enemies of WSS, impeding the biological control of this pest. Cultural practices offer limited benefits and often involve additional costs. WSS management strategies have therefore conventionally relied on host plant resistance (Beres et al. 2011b).

A key trait in host plant resistance to WSS is the pith-filled stem structure that reduces larval survival (Beres et al. 2011a, b; Buteler et al. 2015) and deters oviposition (Varella et al. 2017). Recently, long noncoding RNAs in wheat and microRNAs (miRNAs) in WSS larva were implicated in wheat resistance to infestation and the suppression of this resistance, respectively (Cagirici et al. 2017a). A major locus on chromosome 3BL, *Qss.msub-3BL*, was found to account for at least 76% of the total variation in stem solidness, and other minor alleles located on chromosomes 1B, 3D, and 5D have also been identified in hexaploid wheat (Nilsen et al. 2016; Varella et al. 2015). A single dominant gene, *SSt1*, controlling stem solidness in durum wheat, has been mapped to chromosome 3BL, in the region of the *Qss.msub-3BL* locus (Nilsen et al. 2016). Despite several studies aiming to map the loci responsible for the solid stem phenotype, the underlying molecular mechanisms contributing to this key trait remain elusive.

The annotation and assembly of transcripts from WSS larva and male and female adults revealed the main gene classes involved in ion-binding and biosynthetic processes (Cagirici et al. 2017a). The cross-kingdom regulation of miRNAs was also elucidated to assess the possible effects of larval miRNAs on wheat. A total of 10 putative wheat targets for three larval miRNAs were reported on wheat chromosome 3, including regions encoding the probable methyltransferase PMT11 and ankyrin-like proteins (Cagirici et al. 2017a). These putative wheat targets of larval miRNAs therefore likely function in the wheat defense mechanisms against WSS.

In this study, we used a multiomics approach in the solid- and semi-solid-stemmed hexaploid wheat cultivars, Choteau and Scholar, respectively, to better understand the underlying molecular mechanisms of the WSS response and the contribution of stem solidness to WSS resistance. We also conducted a comparative genomics analysis of the stem solidness loci in the wild emmer (*T*. *turgidum* ssp. *dicoccoides*), durum, and bread wheats, and analyzed the syntenic relationship of the 3BL locus in three close relatives of wheat: barley (*Hordeum vulgare*), Brachypodium (*Brachypodium distachyon*), and rice (*Oryza sativa*). Furthermore, we conducted an in silico miRNA identification for 3BL to gain further insights into the regulatory mechanisms that could contribute to stem solidness and WSS resistance. Taken together, this study represents the first report of the advantages of using comparative genomics, transcriptomics, proteomics, and metabolomics approaches to provide a comprehensive understanding of wheat defense mechanisms against WSS, and paves the way for future improvements in WSS management.

## Materials and methods

### Datasets used in this study

Molecular markers associated with stem solidness were retrieved from previous reports (Nilsen et al. 2017), and marker sequences were retrieved from either TriticeaeToolbox (https://triticeaetoolbox.org/) or GrainGenes (http://wheat.pw.usda.gov/GG3/). The 3B chromosome sequences and transcript annotations used were from wild emmer wheat (*T*. *turgidum* ssp. *dicoccoides* genotype “Zavitan”; Avni et al. 2017) and the 3B chromosome assemblies, or pseudomolecules, and transcript annotations were from bread wheat (*T*. *aestivum* cv. Chinese Spring; IWGSC 2014; Choulet et al. 2014).

### Plant material and WSS treatment

In this study, stem tissues from two wheat cultivars, the solid-stemmed “Choteau” (PI633974) and semi-solid-stemmed “Scholar” (PI607557), were used. These lines carry distinct alleles for solid stems at *Qss.msub-3BL.* All plants were grown in the greenhouse under a 16-h photoperiod. Adult WSS (*Cephus cinctus*) were obtained for the experiments. Three plants per pot were grown with daily watering, incorporating twice-a-week fertilizing after the three-leaf stage. At Zadok stage 32 (two detectable internodes), the plants were individually placed inside infestation chambers containing three adult female WSS for 3–4 days. Control plants were caged but not exposed to WSS. Plant tissues were sampled 14 days after the initial infestation date; first, the lower internodes of the stem were dissected to identify infestation with either eggs or larvae, then the plant material was collected solely from internodes 3 and 4, at the top of the region containing mature tissues. The same stem section was collected from the control plants. Stem material from infested and control plants was immediately flash-frozen in liquid nitrogen and stored at − 80 °C.

### Total RNA isolation, sequencing, and identification of differentially expressed genes

Total RNA was extracted for three replicates of control and infested stem tissues from the two cultivars, as previously described (Cagirici et al. 2017a). RNA sequencing (RNA-Seq) was performed on the isolated RNA samples with RNA integrity numbers of greater than 7. The library was prepared using a TruSeq RNA Library Preparation Kit (Illumina, San Diego, CA, USA), following the manufacturer’s instructions, and sequencing was performed using an Illumina Hi-Seq 2000 platform. The RNA-Seq reads were trimmed using Sickle (v.1.33) before being aligned to the *T*. *aestivum* chromosome 3B assembly using GMAP (v.2017-02-25; Wu and Watanabe 2005). The aligned reads were then compared to a GFF file containing annotated transcripts of *T*. *aestivum* (variety Chinese Spring) and the read counts were obtained using HTSeq-count (−m union; Anders et al. 2015). Raw read counts were filtered using the Noleaven R package (https://github.com/topherconley/noleaven) to eliminate those with zero or low counts across the entire dataset. The identification and functional annotation of differentially expressed genes (DEGs) between the control and WSS-treated stem samples of Choteau and Scholar were performed as previously described (Cagirici et al. 2017a).

### Analysis of proteome changes in response to WSS infestation

The total proteins of three replicates of the control and infested stem tissues of Choteau and Scholar were extracted using the TCA-phenol method (Wang et al. 2006) and quantified as described previously (Esen 1978). The protein extracts were denatured by the addition of lysis buffer (1:1) containing 7 M urea, 2 M thiourea, and 4% 3-((3-cholamidopropyl) dimethyl ammonio)-1-propanesulfonate (CHAPS), followed by the addition of 30 mM Tris-HCl (pH 8.8). Subsequently, each set of three samples, labeled with a CyDye dilution of Cy2, Cy3, or Cy5 (GE Healthcare Biosciences, Pittsburgh, PA, USA), was run on a single gel. The labeling was stopped by the addition of 0.7 μL l-lysine (10 mM) followed by a 15-min incubation at 4 °C. The labeled samples were mixed with an equal volume of 2× 2D sample buffer (8 M urea, 4% CHAPS, 20 mg/mL dithiothreitol (DTT), 2% Pharmalytes (GE Healthcare Biosciences), and a trace amount of bromophenol blue) and 100 μL destreak solution (GE Healthcare Biosciences). The total sample volumes were adjusted to 260 μL with rehydration buffer (7 M urea, 2 M thiourea, 4% CHAPS, 20 mg/mL DTT, 1% Pharmalytes, and a trace amount of bromophenol blue). The samples were analyzed for isoelectric focusing on a 13-cm precast non-linear immobilized pH gradient strip (pH 4–9; GE Healthcare Biosciences) and separated for size in the second dimension using sodium dodecyl sulfate polyacrylamide gel electrophoresis. The gels were scanned using a Typhoon Trioscanner (GE Healthcare Biosciences) following the manufacturer’s protocol, and the gel images were processed with Image Quant (v.5.0; GE Healthcare Biosciences).

The differential protein levels were quantified using a differential in-gel analysis, and a quantitative analysis of the protein spots was performed using DeCyder software (v.6.5; GE Healthcare Biosciences). Quantitative comparisons of spots were performed for samples run at the same time, and the pair-wise volume ratios were calculated for each protein spot to determine the relative protein levels. A Student’s *t* test was conducted using the log_2_-normalized average spot volume ratios for all spots detected from the three replicates of each experiment. Only spots representing differentially regulated proteins with a ≥ 1.35-fold and statistically significant (*p* value < 0.05) difference were selected for mass spectrometry (MS). The selected spots were subjected to in-gel trypsin digestion, peptide extraction, desalting, and spotting on a matrix-assisted laser desorption/ionization (MALDI) plate, followed by a MALDI time-of-flight (TOF) analysis for protein identification. The mass spectra of the peptides in each sample were obtained using an Applied Biosystems Proteomics Analyzer (Thermo Fisher Scientific, Waltham, MA, USA), and the 10–20 most abundant peptides in each sample were further subjected to fragmentation and a tandem MS/MS analysis. Combined results from the MS and MS/MS spectra were submitted for a primary sequence database search using the GPS Explorer software equipped with the MASCOT search engine to identify the proteins. The highest scoring hit from the database search for each 2D gel spot was used as the protein identification label. Candidates with a protein score confidence interval (C.I.) or ion C.I. of > 95% were considered significantly DEGs.

### Analysis of metabolome changes in response to WSS infestation

Total metabolite extraction was performed for three replicates of control and infested stem tissues of Choteau and Scholar. Approximately 150 g of the frozen stem sample was ground in liquid nitrogen and immersed in 100% methanol at 70 °C for 15 min. Subsequently, the samples were vortexed and centrifuged at 25,000*g* for 10 min at 4 °C. The metabolites were separated by acetone precipitation at − 80 °C with overnight incubation, followed by centrifugation at 25,000*g* for 10 min at 4 °C. The resulting metabolite supernatant was dried in a speed vacuum and stored at − 80 °C.

Following the method of Fiehn et al. (2008), the metabolites were separated using gas chromatography (GS)-MS, using helium as the mobile phase with a flow rate of 1 mL min^−1^, on a Rtx-5Sil MS column (30 m length × 0.25 mm internal diameter with a 0.25-μm film of 95% dimethyl/5% diphenylpolysiloxane; Restek Corporation, Bellefonte, PA, USA). The injection temperature was 50 °C, which ramped to 250 °C by 12 °C s^−1^. The initial oven temperature was 50 °C for 1 min, which was then ramped at 20 °C min^−1^ to 330 °C and held constant for 5 min. Following the GC-TOF MS, the primary metabolites were profiled and their abundance was estimated based on peak intensity. Scaling and centering of the data were performed following the method suggested by van den Berg et al. (2006). Metabolites that are produced by the pathways suggested to be differentially regulated under WSS infestation by the transcriptomic and proteomic data were analyzed further.

### In silico miRNA identification, target prediction, and annotation

A total of 1404 non-redundant high-confidence and/or experimentally identified mature miRNA sequences from 72 Viridiplantae species were collected from miRBase (v.21, June 2014; Kozomara and Griffiths-Jones 2011). A two-step homology-based in silico method was then used to identify miRNAs from the transcriptome sequences detailed above, as previously described (Akpinar et al. 2015; Alptekin and Budak 2017; Cagirici et al. 2017b; Kantar et al. 2012; Kurtoglu et al. 2014). Potential mRNA targets of the miRNAs were identified with user-defined query and target options defined by Dai and Zhao (2011).

## Results

### Comparative genomics analysis of stem solidness loci in wild emmer, durum, and bread wheats

**Fig. 1:**
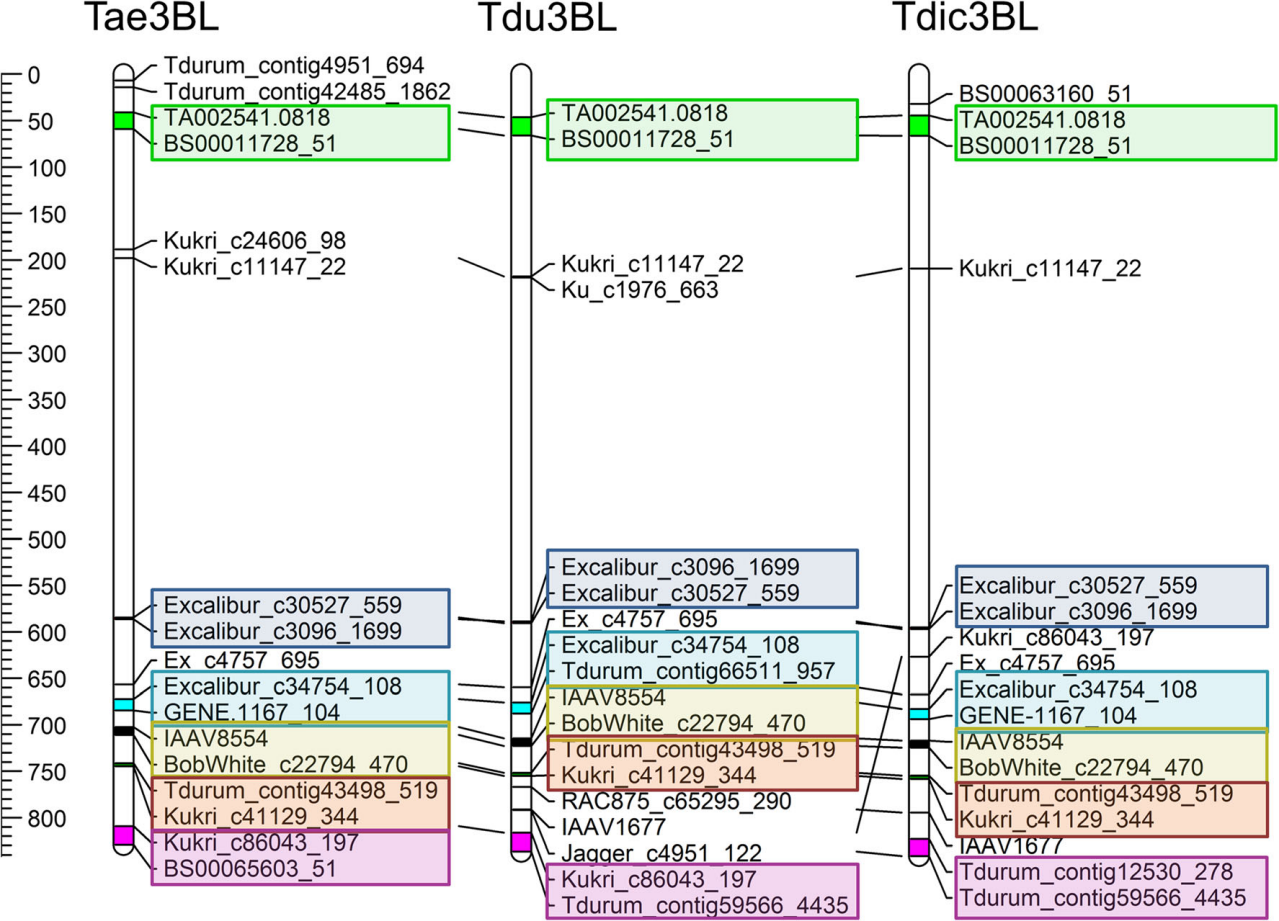
*Comparative analysis of the regions delineated by the molecular markers linked to stem solidness on the 3B chromosomes of T*. *aestivum*, *T*. *turgidum* durum, *and T*. *turgidum* dicoccoides*.* The six regions identified by the molecular markers are indicated by different colors. Tae, *Triticum aestivum* cultivar Chinese Spring; Tdu,: *Triticum turgidum durum* accession MWG; Tdic, *Triticum turgidum dicoccoides* accession Zavitan

Stem solidness, characterized by pith formation inside the stem that serves as a physical barrier against larval movement, is considered to be the most important trait in the integrated pest management of WSS. Although this complex trait appears to be under the control of several genes on different chromosomes, the *Qss.msub-3BL* locus, located on the long arm of chromosome 3B, accounts for most of the variation in wheat stem solidness (Kong et al. 2013; Nilsen et al. 2017). To explore the physical locations of the genetic determinants of stem solidness, including the *Qss.msub-3BL* locus, previously published molecular markers linked to this trait (Nilsen et al. 2017) were mapped to the chromosome 3B pseudomolecules of *T*. *turgidum* ssp. *dicoccoides* genotype Zavitan (wild emmer wheat), *T*. *turgidum* ssp. *durum* genotype MWG (tetraploid durum wheat), and *T*. *aestivum* cv. Chinese Spring (hexaploid wheat; Fig. 1). Wild emmer wheat is a close wild relative of durum wheat, as both diverged from the common ancestor *T*. *turgidum*, the tetraploid progenitor of *T*. *aestivum* (Ani Akpinar et al. 2015). The close relationship between the three wheat genomes appears to be retained for the molecular markers associated with the stem solidness trait (Fig. 1).

**Fig. 2:**
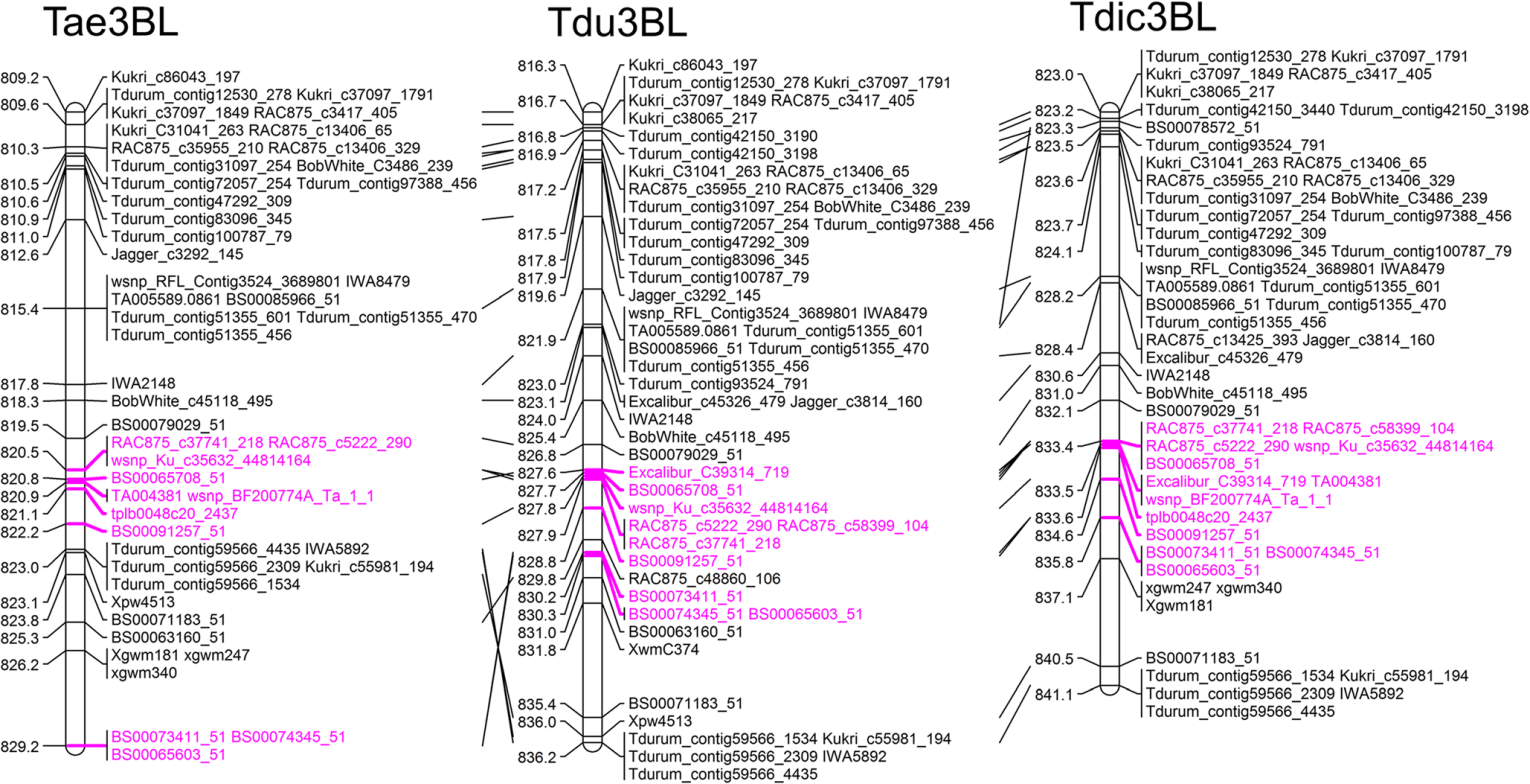
*Comparative analysis of the* Qss-3BL *QTL associated with WSS resistance in T*. *aestivum*, *T*. *turgidum* durum, *and T*. *turgidum* dicoccoides, *indicated by molecular markers.* Pink shading indicates the most probable location of the *SSt1* gene found in tetraploid wheat. Tae, *Triticum aestivum* cultivar Chinese Spring; Tdu, *Triticum turgidum durum* accession MWG; Tdic, *Triticum turgidum dicoccoides* accession Zavitan

The physical location of the molecular markers linked to stem solidness identified six regions associated with this trait along chromosome 3B. The first region was located on the short arm, while the remaining five loci were clustered closer to the distal end of the long arm of chromosome 3B. Besides these regions, five additional markers were mapped to separate locations. Curiously, two of these markers suggested that an additional locus exists in durum wheat within a cluster of 11 molecular markers. Although the molecular markers indicated a scattered positioning of genetic determinants for stem solidness, most were clustered specifically at the distal-most region of chromosome 3B (Fig. 2). This region, consistent with previous reports (Nilsen et al. 2017), likely represents the *Qss.msub-3BL* locus, and is located between 809.2–829.2 Mb (i.e., between markers Kukri_c86043_197 and BS00065603) in hexaploid wheat, 823–841.1 Mb (Tdurum_contig12530_278 and Tdurum_contig59566_4435) in emmer wheat, and 816.3–836.2 Mb (Kukri_c86043_197 and Tdurum_contig59566_4435) in durum wheat. This locus is hereafter referred to as *Qss*-3BL.

### Syntenic analysis of *Qss*-3BL in barley, *Brachypodium*, and rice

**Fig. 3:**
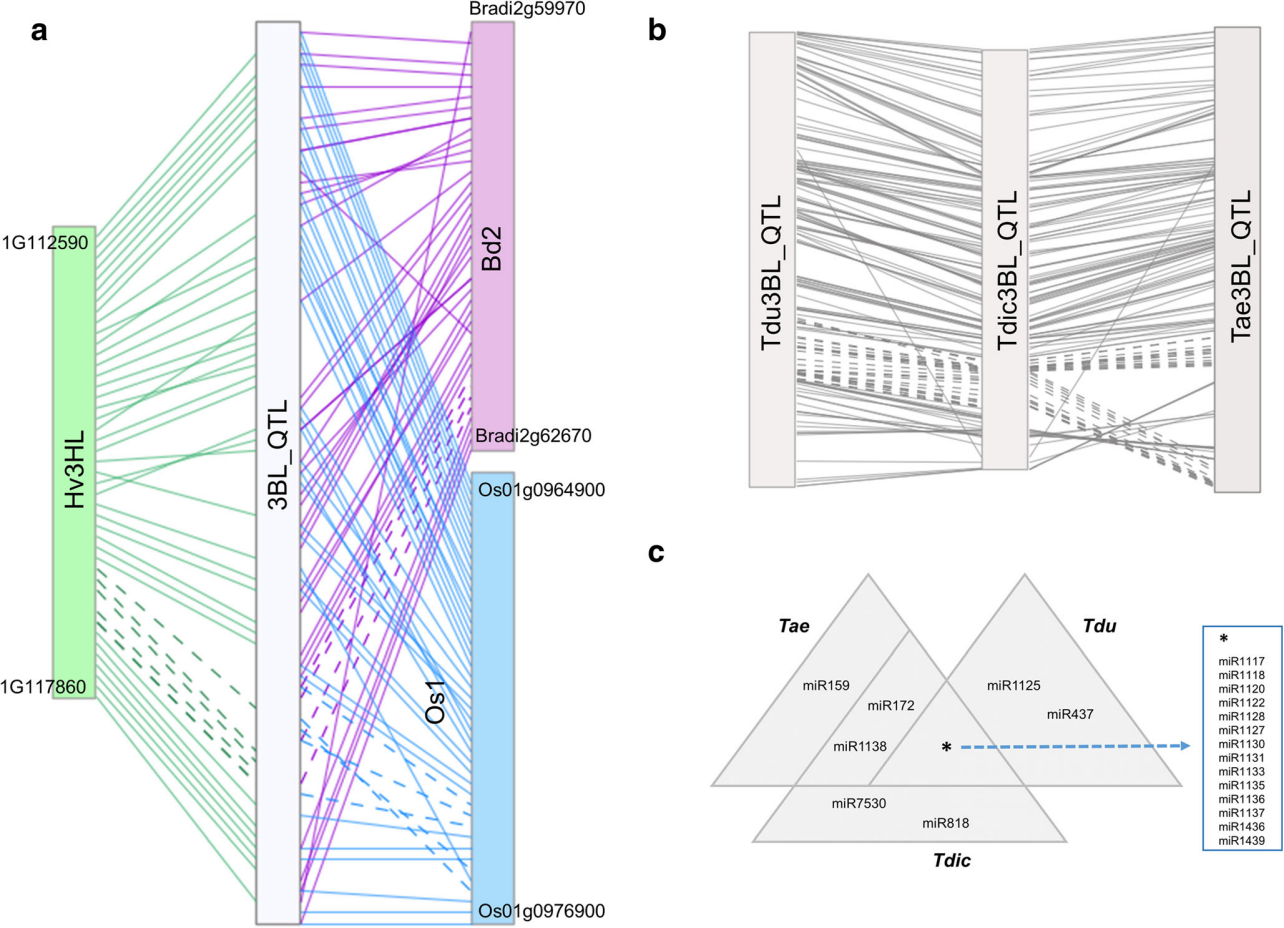
*Chromosomal view of the syntenic relationships of the WSS response locus and the regulatory miRNAs encoded in the* Qss-3BL *QTL*. **a** Locations of *Qss-3BL* QTL in *Brachypodium distachyon* chromosome 2 (Bd2), *Oryza sativa* chromosome 1 (Os1), and *Hordeum vulgare* chromosome 3 (Hv3H). **b** *T*. *aestivum*, *T*. *turgidum durum*, and *T*. *turgidum dicoccoides*. **c** miRNAs identified from the *Qss-3BL* QTL of *T*. *aestivum*, *T*. *turgidum durum*, and *T*. *turgidum dicoccoides*

No information on the conservation of the well-known wheat *Qss-3BL* quantitative trait locus (QTL) is available in barley, *Brachypodium*, and rice, close relatives of wheat, despite the threat that WSS poses to barley production (Portman et al. 2016). This is not surprising, as a comparison of the molecular markers from the *Qss-3BL* QTL with the syntenic *Brachypodium* chromosome 2 (Bd2) and rice chromosome 1 (Os1) indicated little conservation at the nucleotide level. Thus, to explore the syntenic relationships between these closely related species, wheat transcript sequences mapped to the *Qss-3BL* interval were compared against the annotated proteins from barley, *Brachypodium*, and rice. A best reciprocal hit strategy was used, with the sequence similarity cutoff for significant pairs adjusted for barley, the closest wheat relative of the three. This comparison identified orthologous *Qss-3BL* genes in the syntenic chromosomes Bd2, Os1, and the long arm of chromosome 3H in barley (Hv3HL). While there was extensive colinearity between the wheat *Qss-3BL* QTL and barley Hv3HL, the syntenic regions on Bd2 and Os1 indicated a few rearrangements (Fig. 3a). The syntenic regions on Bd2 appeared to span between Bradi2g59970 and Bradi2g62670, encompassing 40 genes, although orthologous genes were identified on more distant regions as well, such as Bradi2g04010. The syntenic block on Os1 was defined by 42 genes located between Os01g0964900 and Os01g0976900, although, similar to *Brachypodium*, more orthologous genes were found in other regions of the chromosome. In barley, the syntenic interval was composed of 44 highly conserved genes between HORVU3Hr1G112590 and HORVU3Hr1G117860, in an almost identical order to the orthologous genes in the wheat *Qss-3BL* locus (Fig. 3a). The high level of conservation for these loci between the 3BL and 3HL chromosomes demonstrates the close evolutionary relationship between wheat and barley. The functional annotations of the *Brachypodium*, rice, and barley genes located on the putative WSS response loci were strikingly similar to the wheat genes (Supplementary Table S1). This observation suggests that, in addition to their considerable colinearity, these loci may also be functionally conserved among the grasses.

Recently, the *SSt1* gene, widely accepted as the basis for stem solidness in tetraploid wheat, was reported to be located between 833.4 and 835 Mb on emmer wheat chromosome 3B (Nilsen et al. 2017). A comparative transcriptomic analysis of the *Qss-3BL* locus between hexaploid bread wheat and the tetraploid wild emmer and durum wheats indicated that the transcripts located in this QTL are highly colinear (Fig. 3b); however, the putative *SSt1* interval within the *Qss-3BL* QTL appears to be split in the hexaploid bread wheat, and the transcripts between these split fragments had no significant counterparts in wild emmer wheat (Fig. 3b, dashed lines). The *SSt1* gene therefore appears to be confined to the tetraploid genotypes.

### miRNAs processed from the *Qss-3BL* locus

Putative miRNAs encoded from the distal-most loci delineated by the *Qss-3BL* molecular markers on chromosome 3B of bread, durum, and wild emmer wheats, including the putative *SSt1* gene reported in previous study (Nilsen et al. 2017), were explored using a two-step homology-based optimized method (Lucas and Budak 2012; Alptekin et al. 2016). The comparison of these regions to the 1404 high-confidence mature miRNA sequences of plants retrieved from the miRBase database (Release 21) indicated that these three loci from bread, durum, and wild emmer wheats carried precursor sequences for 454, 451, and 447 unique mature miRNAs belonging to 17, 18, and 16 families, respectively (Fig. 3c). Of these miRNA families, the miR159 family was only identified in bread wheat, miR1125 and miR437 were only found in durum wheat, while miR7530 and miR818 were specific to loci on the wild emmer wheat 3B chromosome. Furthermore, miR1139 and miR172 were only shared by the bread and durum wheat loci on the 3B chromosomes, not the wild emmer wheat. The remaining 14 miRNA families were common in all three species (Budak et al. 2015).

Plant miRNAs require near-perfect complementation with their target transcripts, enabling the robust identification of potential targets for a given miRNA (Budak and Akpinar 2015). All candidate mature miRNAs were compared against high-confidence coding sequences from bread, durum, and wild emmer wheats to explore the potential miRNA-target pairs and possible networks behind the regulation of the WSS response. miRNAs stemming from the *Qss-3BL* locus in bread wheat targeted 122 coding transcripts, only one of which mapped to the same region. Notably, this single transcript was involved in miRNA-target pairing with 23 unique mature miRNA isoforms from the miR1127 family, even though it did not appear among the DEGs between Choteau and Scholar under control conditions and WSS infestation. Interestingly, 83 mature miRNAs were found to potentially regulate the expression of seven transcripts on the *Qss-3BL* locus believed to encode disease resistance-related proteins. A total of 632 transcripts were potentially targeted by 337 unique mature miRNAs, although only three of them mapped onto the interval potentially containing *SSt1* on durum wheat chromosome 3B. Similar to the transcripts targeted by the bread wheat miRNAs, seven durum wheat transcripts targeted by the miRNAs potentially encoded disease resistance-related proteins. None of the 292 coding targets of wild emmer wheat miRNAs were transcribed from the WSS response locus, whereas four genes encoding disease-resistance proteins in other genomic regions were targeted by four of the *Qss-3BL* miRNA families.

### Differentially regulated transcripts in response to WSS infestation

To gain further insights into how the six 3B loci associated with stem solidness contribute to this trait, RNA-Seq was performed on the semi-solid and solid stem tissues of two bread wheat cultivars, Scholar and Choteau, with and without WSS infestation. In total, 36 transcripts from the six loci were differentially regulated in the different genotypes and/or in response to WSS infestation (*p* value < 0.05).

To identify the differentially regulated transcripts, clean RNA-Seq reads were mapped onto the *T*. *aestivum* Chinese Spring RefSeq v.1 chromosome 3B pseudomolecule. In general, a trend towards downregulation was observed, including for potentially stress-related transcripts (Table 1). The semi-solid-stemmed cultivar Scholar exhibited the differential regulation of 15 transcripts in response to WSS infestation, of which only five were upregulated in comparison with the control conditions. Transcripts encoding components of protein metabolism and transport, including an AP-3 complex subunit beta-2 and an F-box domain-containing protein, as well as multiple peptidases, were downregulated in the infested Scholar stem tissues. In addition, two leucine-rich repeat (LRR) domain-containing proteins, one of which contained a NB-ARC domain, were also downregulated. By contrast, auxin signaling may be promoted in response to WSS infestation, as indicated by the upregulation of a transcript encoding an auxin efflux carrier component. Other transcripts upregulated in response to WSS infestation included those encoding the biotic stress-related constitutive expressor of pathogenesis-related (PR) genes 5 (CPR-5) protein, NADH dehydrogenase (NDH-A), and a magnesium transporter (Table 1).

**Table 1:** Differentially regulated transcripts between semi-solid-stemmed Scholar and solid-stemmed Choteau wheat cultivars, with and without WSS infestation

			ChoInf vs. ChoWT		SchInf vs. SchWT		ChoWT vs. SchWT		ChoInf vs. SchInf	
Transcript	Location^a^ (bp)	Protein product	Log FC	*p* val	Log FC	*p* val	Log FC	*p* val	Log FC	*p* val
TraesCS3B01G069400	41,351,591	Domain of Unknown Function (DUF1618)-containing protein	–	–	–	–	− 4.8	0.020	–	–
TraesCS3B01G069500	41,426,379	–	–	–	–	–	− 2.7	0.044	–	–
TraesCS3B01G072700	43,832,324	Putative LRR receptor-like serine/threonine-protein kinase	–	–	–	–	2.0	0.034	–	–
TraesCS3B01G074900	46,007,493	–	–	–	–	–	–	–	3.3	0.003
TraesCS3B01G076900	47,452,833	Thaumatin domain-containing protein kinase	–	–	–	–	1.8	0.019	–	–
TraesCS3B01G080500	50,932,437	Threonyl-tRNA synthetase	–	–	− 5.6	0.012	–	–	5.7	0.012
TraesCS3B01G086500	54,784,828	Receptor-like protein kinase FERONIA	− 5.1	0.010	–	–	–	–	− 4.5	0.025
TraesCS3B01G088200	56,128,728	Putative disease-resistance protein RGA3	–	–	–	–	–	–	−5.0	0.031
TraesCS3B01G373000	585,829,361	Metalloenzyme superfamily protein/p	–	–	–	–	–	–	1.4	0.011
TraesCS3B01G435600	673,849,549	Rhodanese-like domain-containing protein	− 3.0	0.047	–	–	–	–	− 3.0	0.046
TraesCS3B01G438500	677,740,856	–	–	–	–	–	− 4.8	0.022	–	–
TraesCS3B01G440800	680,420,121	Probable magnesium transporter	–	–	2.7	0.025	–	–	–	–
TraesCS3B01G458900	702,682,149	LRR-domain-containing protein tyrosine kinase	–	–	− 3.4	0.025	–	–	–	–
TraesCS3B01G461200	704,324,107	CPR-5-like protein	–	–	1.2	0.043	–	–	–	–
TraesCS3B01G462800	705,699,031	alpha/beta hydrolase-fold-containing protein	–	–	–	–	–	–	− 1.6	0.034
TraesCS3B01G462900	705,702,223	Auxin efflux carrier component, transmembrane protein	–	–	1.5	0.009	–	–	–	–
TraesCS3B01G466500	708,950,606	Arabinosyltransferase ARAD1/exostosin domain-containing protein	–	–	–	–	–	–	− 4.3	0.035
TraesCS3B01G499400	744,228,112	–	–	–	− 3.8	0.003	–	–	–	–
TraesCS3B01G580400	809,479,669	Salt stress response/antifungal domain-containing protein kinase	− 4.5	0.030	–	–	–	–	–	–
TraesCS3B01G580700	809,602,487	KH domain-containing protein	–	–	–	–	1.8	0.040	–	–
TraesCS3B01G580900	809,625,786	–	− 3.1	0.045	− 4.2	0.006	–	–	–	–
TraesCS3B01G582100	810,450,715	Retrotransposon-related protein	–	–	–	–	2.6	0.005	–	–
TraesCS3B01G582200	810,472,321	NDH-A protein	–	–	2.1	0.017	–	–	–	–
TraesCS3B01G583000	810,948,315	F-box domain-containing protein	–	–	− 3.5	0.016	–	–	–	–
TraesCS3B01G584600	811,457,387	–	–	–	–	–	–	–	− 1.6	0.004
TraesCS3B01G585600	812,569,184	Aminopeptidase I zinc metalloprotease domain-containing protein	–	–	1.9	0.031	–	–	–	–
TraesCS3B01G588300	814,446,746	LRR-domain-containing protein kinase	− 1.8	0.023	–	–	–	–	–	–
TraesCS3B01G589000	814,699,730	Methionine aminopeptidase-like protein	–	–	− 2.5	0.038	–	–	–	–
TraesCS3B01G591700	816,170,822	Wall-associated receptor kinase	− 4.6	0.034	–	–	–	–	–	–
TraesCS3B01G593900	817,408,885	NB-ARC-LRR domain-containing protein	–	–	− 4.6	0.031	–	–	–	–
TraesCS3B01G594400	817,710,500	–	–	–	− 5.4	0.011	–	–	–	–
TraesCS3B01G596400	819,298,156	Cytochrome P450 domain-containing protein/4-hydroxyphenylacetaldehyde oxime monooxygenase-like	− 4.6	0.024	–	–	–	–	–	–
TraesCS3B01G597900	819,930,084	Peptidase C13 family protein	–	–	− 2.3	0.034	–	–	–	–
TraesCS3B01G600100	820,789,324	AP-3 complex subunit beta-2	–	–	− 5.2	0.009	–	–	4.2	0.044
TraesCS3B01G602400	822,535,369	Cysteine-rich receptor-like protein kinase 27	3.6	0.026	–	–	–	–	–	–
TraesCS3B01G611600	829,273,469	Soluble inorganic pyrophosphatase	− 5.1	0.029	–	–	–	–	–	–

*ChoInf*, Choteau infested; *ChoWT*, Choteau wild type; *SchInf*, Scholar infested; *SchWT*, Scholar wild type

^a^Physical position on the *T*. *aestivum* 3B pseudomolecule

The solid-stemmed variety Choteau had nine differentially expressed genes upon WSS infestation, of which only one, encoding a cysteine-rich receptor-like protein kinase, was upregulated. Transcripts potentially encoding stress-related proteins and components of signal transduction, including the receptor-like protein kinase FERONIA, a rhodanese-like domain-containing protein, a salt stress response/antifungal domain-containing protein kinase, an LRR protein kinase, and a wall-associated receptor kinase, were strongly downregulated in Choteau stems in response to WSS infestation (Table 1).

While the comparison of control and infested samples provides insights into the molecular mechanisms selectively activated in response to WSS infestation, a comparison of cultivars with varying levels of stem solidness under both control and infestation conditions may reveal the specific phenotype that restricts WSS larval growth, i.e., stem solidness. A comparison of the Choteau and Scholar stem tissues in control conditions revealed seven transcripts that were differently regulated between these cultivars. Under normal growth conditions, the stem-tissue expression levels of genes encoding an LRR protein kinase, a thaumatin domain-containing protein kinase, and a KH-domain-containing protein were up to twice as high in Choteau than in Scholar (Table 1). By contrast, a transcript encoding a Domain of Unknown Function (DUF)-containing protein and two transcripts with no homology to any known Viridiplantae proteins were expressed between 2.7- and 4.8-fold higher in Scholar than Choteau stems.

In response to WSS infestation, the expression levels of transcripts encoding a threonyl-tRNA synthetase and an AP-3 complex subunit beta-2 were higher in Choteau stems than in Scholar, as these transcripts were strongly downregulated in the Scholar stem tissue upon infestation (Table 1). Another potentially stress-related transcript, encoding phosphoglycerate mutase, was also found at higher levels in infested Choteau stem samples. By contrast, the transcripts for the receptor-like protein kinase FERONIA and a rhodanese-like domain-containing protein were less abundant in WSS-infested Choteau than those in Scholar stems, consistent with their downregulation in Choteau stem samples upon WSS infestation. Intriguingly, a transcript encoding the arabinosyltransferase ARAD1, involved in cell wall organization, was more than fourfold scarcer in the WSS-infested Choteau stems than in the infested Scholar stems.

### Plant defense response against WSS at the proteome and metabolome levels

**Fig. 4:**
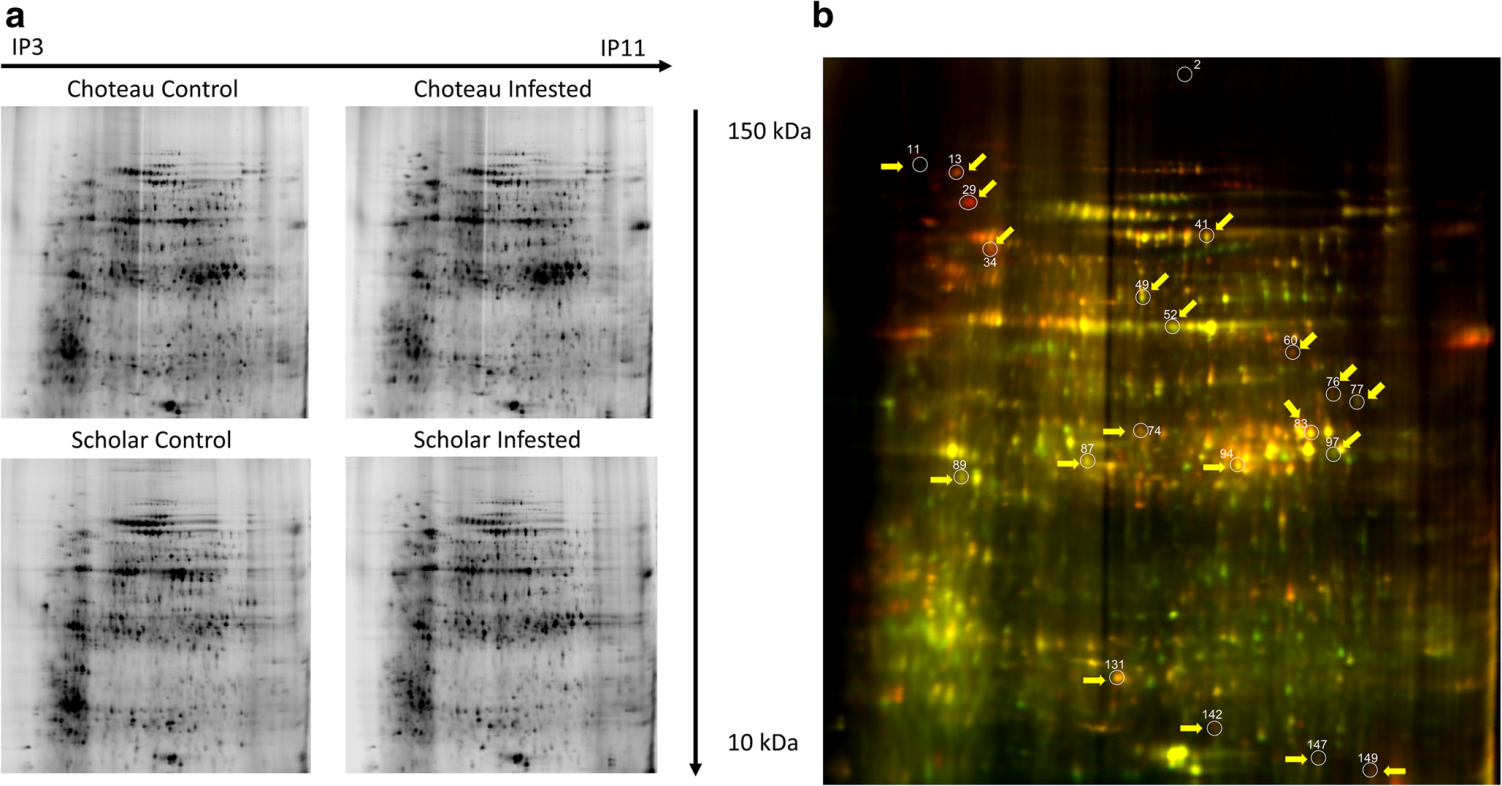
*Proteomics analysis of the WSS response in Scholar and Choteau.* **a** 2D gel photographs of the proteins from the control and WSS-infested stems of Choteau and Scholar. Isoelectronic points (IP) from 3 to 11 and molecular weights (MW) from 150 to 10 kDa are shown on the gel. **b** Statistically significant spots (*p* value < 0.05, fold change > 1.35) chosen for mass spectrometry analysis. All spots are indicated by yellow arrows and white circles

To examine the plant defense response at the proteome level, 2D gel-based proteomics were conducted on stem-tissue samples from Choteau and Scholar. A total of 150 protein spots were detected for both varieties (Fig. 4a), and 21 were differentially regulated under WSS stress and thus selected for protein identification (Fig. 4b, Table 2). Choteau had 10 significantly differently regulated proteins with a *p* value of < 0.05 and a fold change of > 1.35 between the control and infested stems, while Scholar had 15. Four proteins, ureidoglycolate hydrolase (spot no. 77), HMG1/2-like protein (spot no. 149), Ras-related RABB1b (spot no. 131), and glyceraldehyde-3-phosphate dehydrogenase 1 (spot no. 83), were identified as significantly differing between treatments in both varieties (*p* value < 0.05). Some of the proteins that were differentially regulated under WSS, such as ribulose bisphosphate carboxylase, were similar to those that were altered in response to abiotic stress, suggesting their dual role in both abiotic and biotic stress responses (Budak et al. 2013; Lucas et al. 2011). The identified proteins were analyzed in terms of their association with the stem solidness genomic region; a BLAST analysis was used, where a significant association was defined as more than 50% of the alignment length and identity. Six of the 21 proteins were significantly mapped to the genomic regions associated with stem solidness, suggesting that these proteins might be encoded by these loci, while the other five proteins did not map to the *Qss-3BL* region (Table 2). Other proteins showed a level of association to the stem solidness genome regions; however, more research is needed to determine the exact genomic location encoding these proteins.

**Table 2:** Differentially regulated proteins between semi-solid-stemmed Scholar and solid-stemmed Choteau wheat cultivars, with and without WSS infestation

					ChoInf/ChoWT		SchInf/SchWT	
Spot ID	Protein name	NCBI ID	Protein MW (kDa)	Mapped onto stem solidness locus	*p* value	Av. ratio	*p* value	Av. ratio
2	Phenylalanine ammonia-lyase	PAL1_ORYSJ	75,451	Yes, non-significant	0.045	− 1.66	0.49	− 4.37
60	Serine hydroxymethyltransferase 4	GLYC4_ARATH	51,685	No	0.043	2.045	0.11	1.9
76	5-methyltetrahydropteroyltriglutamate--homocysteine methyltransferase 1	METE1_ORYSJ	84,532	Yes, non-significant	0.036	1.605	0.31	− 1.715
83	Glyceraldehyde-3-phosphate dehydrogenase 1, cytosolic	G3PC1_HORVU	36,491	Yes, non-significant	0.026	1.465	0.029	1.83
97	Ribulose bisphosphate carboxylase (large chain)	RBL_HORVU	53,045	Yes, significant	0.012	− 1.535	–	–
142	ATP synthase subunit alpha, mitochondrial	ATPAM_MAIZE	55,146	Yes, non-significant	0.018	1.785	–	–
147	5-methyltetrahydropteroyltriglutamate--homocysteine methyltransferase 1	METE1_ORYSJ	84,532	Yes, non-significant	0.048	1.88	0.34	− 1.56
149	HMG1/2-like protein	HMGL_WHEAT	17,204	No	0.016	1.51	0.048	2.00
11	Putative LRR disease-resistance protein/transmembrane receptor kinase PS19	PS19_PINST	870	No	0.3	1.72	0.026	2
13	Endoplasmin	ENPL_HORVU	92,859	Yes, significant	0.57	2.27	0.043	2.44
29	Heat shock protein 81-1	HSP81_ORYSJ	80,144	Yes, significant	0.39	2.20	0.049	5.96
34	Heat shock cognate 70 kDa protein	HSP7C_PETHY	71,182	No	0.53	0.725	0.03	3.255
41	Putative F-box/FBD/LRR-repeat protein	FDL48_ARATH	48,797	Yes, significant	–	–	0.019	1.99
49	4-hydroxy-7-methoxy-3-oxo-3,4-dihydro-2H-1,4-benzox azin-2-yl glucoside beta-D-glucosidase 1a, chloroplast	HGL1A_WHEAT	64,467	Yes, non-significant	0.73	− 1.36	0.0019	1.90
52	Ribulose bisphosphate carboxylase (large chain)	RBL_WHEAT	52,817	Yes, significant	0.23	− 1.92	0.0056	− 1.85
74	UDP-glucose 6-dehydrogenase 4	UGDH4_ORYSJ	52,821	Yes, non-significant	0.2	1.43	0.00094	1.63
77	Probable ureidoglycolate hydrolase	UAH_ORYSJ	51,683	Yes, non-significant	0.026	− 1.59	0.046	− 2.02
87	Tricetin 3′,4′,5′-O-trimethyltransferase	FOMT2_WHEAT	38,545	Yes, non-significant	0.9	0.18	0.042	− 1.85
89	Fructokinase-2	SCRK2_ORYSJ	35,494	No	0.95	0.04	0.018	− 2.08
94	Serine acetyltransferase 4	SAT4_ARATH	38,400	Yes, significant	0.21	1.695	0.013	1.605
131	Ras-related protein RABB1b	RAB1B_ARATH	23,161	Yes, non-significant	0.07	1.64	0.00	1.54

*ChoInf*, Choteau infested; *ChoWT*, Choteau wild type; *MW*, molecular weight; *SchInf*, Scholar infested; *SchWT*, Scholar wild type

For Choteau and Scholar, respectively, only three of 10 and four of the 15 differentially abundant proteins were downregulated during the WSS treatment, suggesting a general upregulation in protein expression under WSS infestation (Table 2). Across the four significantly differentially produced proteins identified in both varieties, only ureidoglycolate hydrolase (spot no. 77) was downregulated, while the HMG1/2-like protein (spot no. 149), Ras-related RABB1b (spot no. 131), and glyceraldehyde-3-phosphate dehydrogenase 1 (spot no. 83) were upregulated in both varieties.

**Fig. 5:**
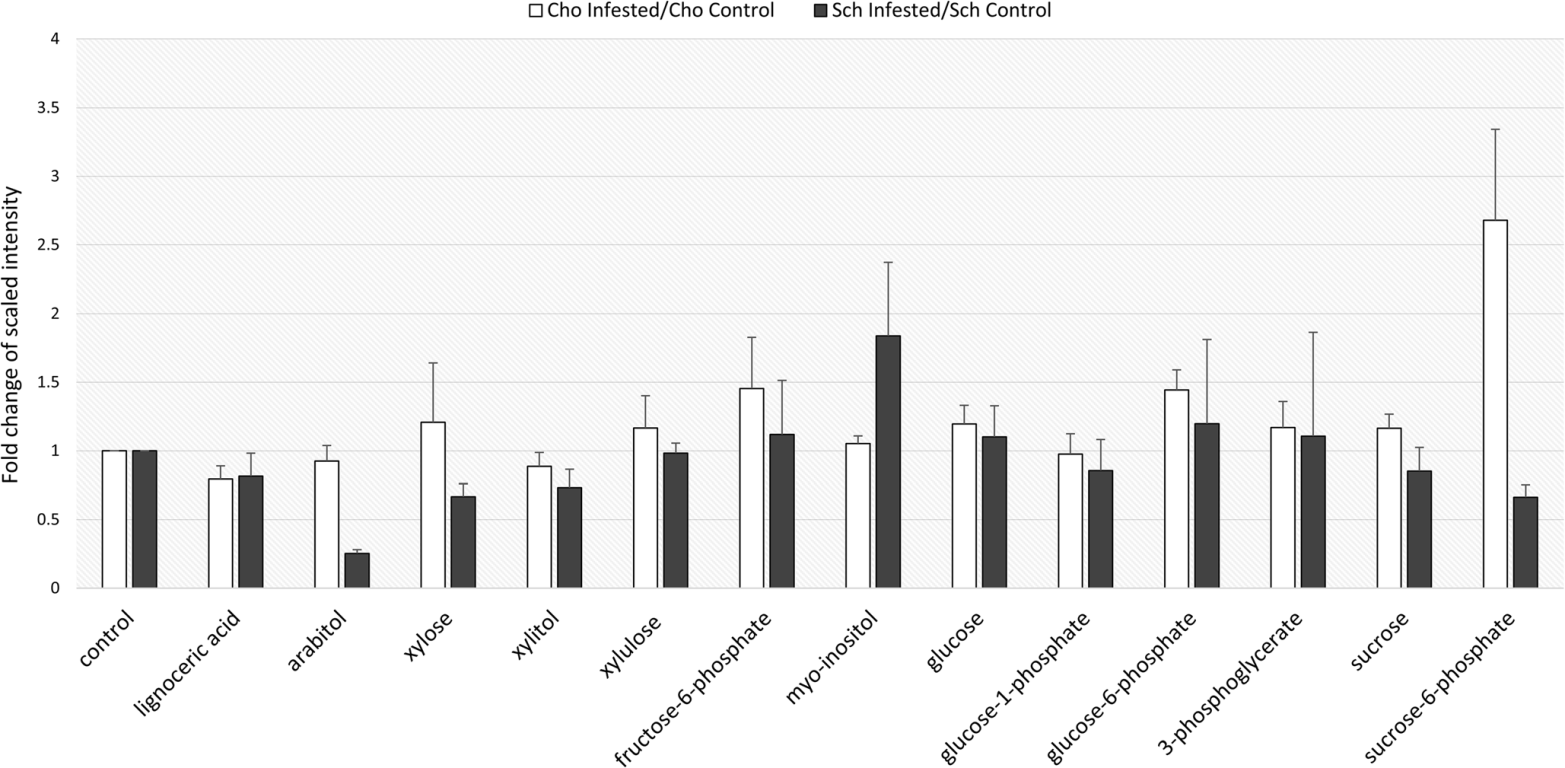
*Differentially regulated metabolites during WSS infestation.* The differential regulation of metabolites belonging to the phenylpropanoid and pentose phosphate pathways, suggested as key pathways. The control for each metabolite is represented as 1, while the fold changes for each metabolite were calculated by dividing the average scaled intensity value of the WSS-stressed samples by that of the WSS-control metabolite samples. Error bars were calculated by the basic rules of error propagation to estimate the average error in each sample

Based on the differential regulation of the proteins and stem solidness-associated transcripts, the phenylpropanoid and pentose phosphate pathways were determined to be highly responsive to WSS infestation at the metabolic level. The regulation of the primary metabolites associated with these pathways was therefore investigated during WSS infestation using a GC-MS analysis. The metabolomic data indicated that the induced decrease in the amount of the enzyme phenylalanine ammonia-lyase (PAL), the initiator of the phenylpropanoid pathway for secondary metabolite production, during WSS infection affected lignin formation in the wheat, indicated by the slight decrease in lignoceric acid detected in the stem tissues of both Choteau and Scholar (Fig. 5).

By contrast, the metabolites from the pentose phosphate pathway were found to be differently regulated in the solid-stemmed Choteau and the semi-solid-stemmed Scholar cultivars. Metabolites associated with the structural sugars arabinose and xylose, which contribute to cell wall biosynthesis, were downregulated in Scholar following WSS infestation, while no significant change was observed in Choteau. Despite the twofold decrease in the amount of fructokinase-2 in Scholar following WSS infestation, there was no significant difference in the abundance of fructose-6-phosphate, the primary compound generated by the activity of this enzyme. The increase in the amount of UDP-glucose 6-dehydrogenase 4 in Scholar did not result in a significant effect on the downstream products of UDP-glucose, such as glucose-1-phosphate and glucose-6-phosphate; however, this may be explained by the significant increase in the amount of myo-inositol, which indicated the oxidation of glucose-6-phosphate to generate pentose sugars. In addition, a strong increase in the amount of sucrose-6-phosphate was observed in Choteau, which may be associated with an upregulation in sucrose production to provide energy for the plant. By contrast, the levels of both sucrose and sucrose-6-phosphate were decreased in Scholar, which may result in a lack of energy and poor defense against WSS.

## Discussion

Although a major locus (*Qss.msub-3BL*) and a single dominant gene in durum wheat (*SSt1*) associated with stem solidness have been identified, in addition to a few minor alleles (Nilsen et al. 2017; Varella et al. 2015), little is known about the molecular mechanisms underlying stem solidness and the alternative types of host plant resistance that may arise from the same locus. Here, we employed a multiomics approach, together with the molecular markers that define the stem solidness-associated locus on chromosome 3BL, to explore the basis of stem solidness and its contribution to WSS resistance, and to investigate the WSS-infestation response in wheat at the molecular level.

### Colinearity of the *QSS.msub-3BL* locus in wheat

Molecular markers genetically mapped to the *Qss.msub-3BL* locus in previous reports were clustered around six separate physical regions on the chromosome 3B sequences of three closely related wheat species, hexaploid bread wheat (Chinese Spring), tetraploid durum wheat (MWG), and tetraploid wild emmer wheat (Zavitan). While one of these physical regions was located on the short arm of the chromosome, the remaining five were consecutively located on the long arm in close proximity to each other. Although these six regions were consistently observed on the 3B chromosomes of all three wheats, an additional locus was identified on durum wheat chromosome 3B; this 217–219-Mb region consisted of 11 clustered markers, a few of which mapped to independent locations in bread wheat and wild emmer wheat (Fig. 1). Consistent with previous reports on the location of the dominant gene *SSt1* either at or near the 3BL QTL in durum wheat, the molecular markers confining *SSt1* to a ~ 1.6-Mb interval identified by Nilsen et al. (2017) also defined a narrow interval within the most telomeric physical regions of all three species (Fig. 2). These telomeric regions, likely representing the major *Qss.msub-3BL* locus, are referred to as *Qss-3BL*.

Despite close evolutionary relationships, the regions syntenic to the *Qss-3BL* locus in *Brachypodium*, rice, and barley have not been accurately defined, likely because of the lack of sequence conservation in the molecular markers, at least between wheat, *Brachypodium*, and rice. Using a more inclusive approach, a comparison of wheat transcripts located within the *Qss-3BL* locus to the protein sequences of the related grasses clearly identified syntenic regions on the *Brachypodium* Bd2 chromosome, the rice Os1 chromosome, and the long arm of the 3H chromosome in barley (Hv3HL; Fig. 3a). The syntenic intervals indicated small-scale inversions, with few perturbations to the synteny of the independent genes. In contrast to the extensive colinearity in the putative *SSt1* region (Fig. 3a, dashed lines) in wheat, barley, and *Brachypodium*, the corresponding region in rice appears to be rearranged. Importantly, a transcriptomic comparison of the *Qss-3BL* locus on the 3B chromosomes of the bread, durum, and wild emmer wheats also indicated a disruption in colinearity, specifically in the putative *SSt1* region in bread wheat (Fig. 3b, dashed lines). This observation was supported by the fact that *SSt1* has only been identified in durum wheat so far (Beres et al. 2013). This split alignment between the bread wheat and wild emmer wheat *Qss-3BL* loci may indicate a rearrangement that occurred after the hybridization of *Aegilops tauschii* with tetraploid *T*. *turgidum* to give rise to the hexaploid *T*. *aestivum*, which somehow resulted in the loss of *SSt1* in the hexaploid genotypes.

### miRNA repertoire of *QSS.msub-3BL* from wild and modern tetraploid and hexaploid wheats

Putative miRNAs from the Qss-3BL locus from bread, durum, and wild emmer wheats included 454, 451, and 447 unique mature miRNA sequences belonging to 17, 18, and 16 miRNA families, respectively, of which 14 were common to all three species (Fig. 3c). The miR1127 family from the *Qss-3BL* locus in bread wheat targeted a single transcript from the same region, pointing to a potential auto-regulatory circuit (Supplementary Table S2a); however, this family is not well characterized in plants, and its target transcript does not exhibit homology to any known plant protein, so the functional aspects of such a circuit could not be determined. Similarly, three miRNA families from the durum wheat *Qss-3BL* QTL, miR1135, miR1436, and miR1439, targeted three transcripts in the same region with no known homologs in the Viridiplantae (Supplementary Table S2b). In contrast to bread and durum wheat, none of the predicted miRNAs from the wild emmer wheat *Qss-3BL* QTL targeted transcripts within this region.

Both the miR1127 and miR1436 families also targeted transcripts outside the *Qss-3BL* locus, including those involved in disease responses (Supplementary Table S2c). Small RNA interactions with putative disease-response genes were also found in the model grass species, *Brachypodium* (Lucas et al. 2014). Other putative miRNAs from the *Qss-3BL* QTLs in the three species also targeted stress-response-related transcripts encoded outside of this locus. Six miRNA families from the bread wheat *Qss-3BL* QTL interacted with seven transcripts encoding disease-resistance proteins, while five miRNA families from the modern and wild tetraploid wheat *Qss-3BL* loci each targeted seven transcripts within their transcriptomes, including disease-resistance-related transcripts. Notably, of all the miRNAs targeting disease-related transcripts, only the miR1117 and miR1439 families were common to all three species. Although these observations imply a link between the *Qss-3BL* QTL and miRNA-mediated regulation of certain stress response pathways, the functions and/or canonical target(s) of these miRNA families are largely unknown, complicating the elucidation of their regulatory pathways. The further characterization of wheat miRNAs and transcriptomes may unravel new dimensions to the WSS response that can be used in pest management and pesticide development.

### Differential regulation of *QSS.msub-3BL*-associated transcripts under WSS infestation

DEGs located within these six physical regions were, in general, downregulated in response to WSS infestation, both in Choteau and Scholar, including genes potentially encoding proteins related to the stress response, cell signaling, protein turnover, and translation machineries, hinting at the metabolic events taking place in the plants. A total of 36 transcripts from the six physical regions defined by the *QSS.msub-3BL* molecular markers showed differential expression patterns in four pair-wise comparison backgrounds: (1) Choteau-infested vs. control, (2) Scholar-infested vs. control, (3) Choteau-control vs. Scholar-control, and (4) Choteau-infested vs. Scholar-infested. The most dramatic change was observed in the infested vs. control Scholar stems, where 15 transcripts were differentially regulated. Since the semi-solid stems of the Scholar likely provide less resistance to larval growth, WSS infestation may be expected to trigger more dramatic changes in the metabolism of this cultivar; however, infestation appears to impede the biotic stress-related cellular processes. For instance, *CPR-5* was upregulated in the Scholar stems upon infestation. CPR-5 is a plant response regulator that inhibits the production of PR proteins and salicylic acid, as well as the accumulation of reactive oxygen species, in the absence of biotic stress, reducing the cost of fitness (Rojas et al. 2014; Vos et al. 2013). Another transcript upregulated upon infestation encodes an aspartyl aminopeptidase, which has been linked to increased susceptibility to bacterial pathogens, as the silencing of this transcript resulted in delayed disease symptoms and decreased host cell death in *Nicotiana benthamiana* (Bae et al. 2013). On the other hand, another transcript encoding a different type of aminopeptidase, methionine aminopeptidase, was downregulated in infested Scholar stems. Although its role in the biotic stress response has not yet been reported, methionine aminopeptidase is an abiotic stress-related protein that can confer cold and freezing tolerance and aid in post-translational modifications (Jeong et al. 2011). Among the downregulated transcripts were two encoding LRR domain-containing proteins, one of which contained an NB-ARC domain as well. LRR-containing receptor kinases are well-known transmembrane proteins that recognize pathogen elicitors and activate the response pathways in the host plant (Huot et al. 2014). Similarly, the NB-ARC-LRR-type proteins are inducers of effector-triggered immunity in plants, as they sense the attack and turn on the response pathways (Takken and Goverse 2012). Moreover, two genes encoding enzymes responsible for the induction of vacuolar enzymes and defense-related molecules, including PR proteins and phytoalexins (Hatsugai et al. 2010), were also significantly downregulated in infested Scholar stems. While it is possible that, upon WSS infestation, Scholar stem cells may shut down mechanisms that would otherwise confer resistance against other stress factors to conserve energy, it could also be argued that the WSS eggs or larvae that managed to survive within the stem may manipulate the host defense systems in favor of their own survival. In addition to its already relatively less solid stem structure, the metabolism of Scholar appears to be highly vulnerable to WSS.

In the Choteau cultivar, a single transcript encoding a cysteine-rich receptor kinase was upregulated in response to WSS infestation, suggesting the activation of cell signaling pathways; however, similar to Scholar, the expression of a transcript encoding an LRR domain-containing protein kinase was repressed in the infested Choteau stems. Another strongly downregulated transcript upon WSS infestation encoded the FERONIA protein, which contains a malectin domain and belongs to a receptor kinase family. FERONIA is believed to be a sensor of cell wall integrity, playing a role in responding to cell wall disruption, such as wounding by herbivores (Savatin et al. 2014; Shih et al. 2014). WSS infestation therefore appears to suppress a number of stress-response pathway proteins in Choteau (Table 1, Supplementary Table S3), as was also observed in Scholar. Indeed, in several species, elicitors derived from insects or their symbiotic bacteria can inactivate or reduce the defense responses that would otherwise be triggered by herbivore chewing or egg deposition in the host plant, improving the herbivore’s chance of survival (Bruessow et al. 2010; Chung et al. 2013; Hogenhout and Bos 2011). Nevertheless, the solid stems of Choteau still conferred a substantial level of tolerance against WSS in this cultivar.

Notably, under the control conditions, three of the four *Qss-3BL* transcripts encoding potential stress-related proteins were more abundant in Choteau than Scholar. These transcripts encoded an LRR-domain-containing serine/threonine kinase, which is a transmembrane receptor with roles in sensing herbivore attack and activating the intracellular response pathways (Huot et al. 2014), a protein kinase with a thaumatin domain classified as a PR protein due to its activation in response to pathogen and insect attacks (Liu et al. 2010), and a K homology domain-containing protein. By contrast, one transcript, encoding a DUF1618 family protein, was less abundant in Choteau than Scholar. The DUF1618 family was recently found to be involved in the abiotic and biotic stress responses in rice, as several family members were repressed by salicylic acid, a key hormone that triggers plant stress responses (An and Mou 2011; Wang et al. 2014). The lower abundance of a DUF1618 family protein in Choteau stems under control conditions is therefore consistent with an elevated level of defense compared with Scholar, even in the absence of the pest. Taken together, the activity of defense-related molecules in Choteau stems in comparison with Scholar may suggest a constitutively higher level of defense under normal growth conditions, stemming from the loci associated with WSS resistance. Upon infestation, the Choteau cultivar may benefit from the physical barrier provided by its solid stem, as well as its basal level of stress-response factors activated in advance, and may therefore be able to provide a quicker response and better resistance to oviposition or larval growth.

During WSS infestation, 10 DEGs were identified between the Choteau and Scholar cultivars. Infested Choteau stems contained more of a transcript encoding a 2,3-bisphosphoglycerate-independent phosphoglycerate mutase than Scholar, which provides pyruvate to mitochondria in the glycolysis pathway to generate energy. Notably, a phosphoglycerate mutase was also upregulated in *Arabidopsis thaliana* in response to insect feeding (Zhang et al. 2010). A threonyl-tRNA synthetase-encoding transcript was almost sixfold more abundant in Choteau than Scholar, which may indicate highly active translation machinery in the cells of the infested Choteau stems. On the other hand, infested Choteau stems contained fewer transcripts encoding potential players in the biotic and abiotic stress tolerance of wheat, such as the receptor-like protein kinase FERONIA, the arabinosyltransferase ARAD1, the disease-resistance protein RGA3, and a rhodanese-like domain-containing protein. Like FERONIA, ARAD1 is associated with the cell wall; the cell walls of *arad1* mutants contain little arabinan, implying a role for ARAD1 in regulating the abundance of this important cell wall component (Sørensen et al. 2006). Rhodanase detoxifies reactive oxygen species that accumulate as a result of stress conditions, thereby protecting cellular compartments (Most and Papenbrock 2015). While the lower levels of transcripts encoding these proteins in infested Choteau stems compared with infested Scholar stems is intriguing, it is possible that solid stems of Choteau are less affected by the restricted larval growth and/or the Choteau stems may downregulate all non-specific stress responses to conserve energy.

### Comprehensive proteomic and metabolomic analysis of the WSS response

Our proteomics study suggested the differential regulation of 21 proteins, of which four were significantly differently produced following WSS infestation in both varieties (Table 2), suggesting an essential common role in the WSS response. HMG1/2-like protein was upregulated in both Choteau and Scholar. HMG proteins are associated with several important nuclear functions, such as DNA repair, recombination, and chromatin remodeling, through their interaction with histone proteins. Studies of Arabidopsis plants under abiotic stress indicated that epigenetic modifications arising from the activity of HMG proteins can play an important role in the response to stresses such as drought and cold (Cusanelli and Chartrand 2015; Kim et al. 2015; Štros et al. 2007). Both proteomic and transcriptomic data suggested the involvement of NBS-LRR-associated disease-resistance proteins in the WSS response, and the expression of such genes might be regulated by the activation of specific stress-response regions of the genome by the HMG proteins. Thus, manipulation of the HMG1/2-like protein might result in the activation of chromosomal sites specific to the insect response following WSS infestation, which would enable the plant to express certain genes involved in defense mechanisms.

Our proteomics analysis also revealed significant changes in the abundance of heat shock proteins (HSPs) and heat shock cognates (HSCs), important molecular chaperones responsible for protein folding, assembly, and degradation during many different cellular processes (Park and Seo 2015). In plants, they also contribute to the activity of the pathogen recognition receptors (PRRs) by aiding their accumulation for the activation of further stress responses (Nekrasov et al. 2009; Park and Seo 2015). HSP70/HSC70 was shown to play a particularly crucial role in the biotic stress response through its involvement in the hypersensitive response (HR; Kanzaki et al. 2003; Park and Seo 2015). The significant accumulation of HSP81-1/HSC70 under WSS stress in Scholar may facilitate the recognition of WSS-specific PRRs and the activation of the HR. On the other hand, HSP81-1/HSC70 might aid protein folding and the elimination of misfolded proteins to protect cellular integrity under WSS stress. The increase in endoplasmin, a member of the HSP90 family that aids protein folding in the endoplasmic reticulum, also suggested changes in protein metabolism and the level of unfolded proteins. Endoplasmin production was also upregulated in response to powdery mildew infection, which further supports its involvement in the biotic stress response (Gupta and Tuteja 2011). Overall, these results suggest that the semi-solid cultivar Scholar is highly affected at the molecular level by WSS larva infestation, which threatens its cellular homeostasis, whereas the solid-stemmed Choteau cultivar is more likely to resist the effects of the infestation.

At the proteomic level, several proteins possessing enzymatic function were differentially regulated, suggesting their further involvement in metabolomic pathways. One of these, a probable ureidoglycolate hydrolase, was significantly downregulated in both Choteau and Scholar under WSS stress. This enzyme functions in nitrogen metabolism by hydrolyzing ureidoglycolate into glyoxylate, carbon dioxide, and two other ammonia products (Li et al. 2015). The regulation of ureide metabolism by the activity of ureidoglycolate hydrolase results in the accumulation of allantoin and allantoate, primary ureide products that accumulate in response to several abiotic stresses (Li et al. 2015; Watanabe et al. 2014). The reduced levels of ureidoglycolate hydrolase under WSS stress might be associated with the accumulation of primary ureide products such as allantoin, which could be used by the plant to generate a stronger signal to further enhance its metabolism for survival.

Furthermore, the proteomic and metabolomic data suggested changes in the phenylpropanoid pathway through the significant downregulation of PAL in solid-stemmed Choteau. The phenylpropanoid pathway is involved in the production of many important secondary metabolites, such as flavonoids and lignin. Lignin protects plants against mechanical damage in stress conditions, including drought or wounding (Vogt 2010), as it is an essential constituent of the secondary cell wall that reinforces vessel hydrophobicity and fibers and provides a physical barrier against pathogens (Vélez-Bermúdez et al. 2015). Under WSS stress, the regulation of the phenylpropanoid pathway is expected to occur in hexaploid wheat as a means of increasing physical support in the stems; however, the downregulation of the PAL enzyme suggests a reverse manipulation of this pathway. To gain a deeper understanding of lignin biosynthesis regulation under WSS stress, we examined the metabolic byproducts of the phenylpropanoid pathway in Scholar and Choteau. A slight decrease in lignoceric acid was detected in both varieties, which suggests that lignin content decreased coincidentally with the decrease in PAL detected at the proteomic level. The wheat stem lignin and cellulose contents and their effect on WSS lodging have already been analyzed in several papers; however, no significant correlation between these molecules and lodging resistance was identified in wheat (Kong et al. 2013). A study in maize (*Zea mays*) suggested a correlation between lignin content and brittle snap stalk breakage (Li 1997), which could indicate that hexaploid wheat might regulate its lignin content to decrease the risk of stalk breakage on the sides of stem thinned by larval feeding activity. Since the mechanical resistance of the stem tissue restricts larval development and feeding, the WSS larvae might generate chemicals that inhibit the production of PAL and secondary metabolites such as lignin, interfering with the plant stress response to increase their chances of survival. The significantly reduced levels of PAL in solid-stemmed Choteau particularly support this hypothesis, since this pith-filled variety restricts the space available for larval growth and reduces their survival; however, further experiments, particularly involving larval enzymes and metabolites, are required to fully comprehend the contribution of the phenylpropanoid secondary metabolite pathway to the WSS response.

Another significantly downregulated phenylpropanoid-pathway enzyme in Scholar under WSS stress was tricetin 3′,4′,5’-O-trimethyltransferase, which catalyzes a methyl transfer reaction using the flavone tricetin (5,7,3′,4′,5′-pentahydroxyflavone) as a substrate to generate 3′-monomethyl-3′,5′-dimethyl-(tricin), and 3′,4′,5′-trimethyl-ether derivatives (Moheb et al. 2013). In rice, tricin appears to act as a natural biocide; the accumulation of tricin had an anti-feeding and anti-oviposition effect on a rice insect pest, the brown planthopper (*Nilaparvata lugens*; Bing et al. 2007), and it provided a fungicidal effect against several fungal diseases of this cereal, including rice seedling rot disease (Kong et al. 2004, 2010). The observed decrease in the amount of tricetin 3′,4′,5′-O-trimethyltransferase suggests a reduced level of tricin, which would further support the feeding of the larvae and oviposition in the Scholar plants. This enzyme has also been associated with lignin biosynthesis because of its ability to react with 5-hydroxyferulic acid. Moheb et al. (2013) found that tricetin 3′,4′,5′-O-trimethyltransferase accumulated despite the declining tricin levels in plants under cold stress, suggesting its secondary role in lignin biosynthesis that could support secondary cell wall production. Thus, the downregulation of tricetin 3′,4′,5′-O-trimethyltransferase will affect both tricin and lignin biosynthesis, promoting larval growth inside Scholar stems.

Secondary metabolite pathway associated with PAL was also found to be differentially regulated in Scholar upon WSS infestation; 4-hydroxy-7-methoxy-3-oxo-3,4-dihydro-2H-1,4-benzoxazin-2-yl glucoside beta-d-glucosidase 1a (DIMBOA glucoside beta-d-glucosidase), which is responsible for the conversion of DIMBOA glucoside to DIMBOA and d-glucose (Sue et al. 2006), was significantly downregulated in Scholar stems. DIMBOA is a well-known toxic compound produced by many cereals as a defense against herbivores, including aphids, rootworms, and caterpillars (Martos et al. 1992; Niemeyer 2009; Song et al. 2011). Feeding the European corn borer (*Ostrinia nubilalis*), an important pest of maize, with different concentrations of DIMBOA increased larval mortality and delayed pupation in a dose-dependent manner (Campos et al. 1989). In many other studies, DIMBOA was shown to be detrimental to aphids fed with artificial media containing this compound, and aphids preferred leaves with a lower DIMBOA content (Wouters et al. 2016). The downregulation of the DIMBOA glucoside beta-d-glucosidase enzyme suggests a decrease in the content of DIMBOA in the Scholar stems that would further promote larval feeding inside these tissues. This may arise from the interference of the WSS larvae with the WSS response in these plants, which was also suggested by the observation of their effects on lignin biosynthesis.

Another metabolomic pathway that may be differentially regulated under WSS stress was the pentose phosphate pathway, which supports both energy metabolism via glycolysis and the formation of structural sugars such as xylose and arabinose for further mechanical support. Glyceraldehyde-3-phosphate dehydrogenase 1, which is required for the conversion of glyceraldehyde-3-phosphate to 1,3-bisphosphoglycerate during glycolysis, was significantly upregulated in both Choteau and Scholar, together with a slight increase in the amount of 3-phosphoglycerate, another metabolite produced during glycolysis. This supports the previous suggestion that WSS infestation increases the energy metabolism of the plant (Muñoz-Bertomeu et al. 2010). Additionally, a significant increase in glucose-6-phosphate and fructose-6-phosphate was observed in both cultivars, particularly Choteau; however, no significant change in the amount of glucose was detected. In Scholar, the level of UDP-glucose 6-dehydrogenase 4 was increased by WSS infestation, while the level of fructokinase-2 was halved, suggesting several regulatory effects on the glucuronic acid pathway during herbivore attack. In this pathway, glucose-6-phosphate is first converted to glucose-1-phosphate, then UDP-glucose is formed and converted by UDP-glucose 6-dehydrogenase into UDP-glucuronic acid, a precursor of four structural sugars involved in cell wall biosynthesis, xylulose, xylitol, xylulose, and arabinose (Reboul et al. 2011). In accordance with the proteomic data, a decrease in the amount of xylose was detected in Scholar, while the xylitol and xylulose levels were slightly affected by WSS infection in both Scholar and Choteau. By contrast, a significant decrease was detected in the abundance of arabitol, the alcohol form of arabinose, in Scholar, but not in Choteau. Based on this observation, it is tempting to propose that the semi-solid stems of Scholar and the solid stems of Choteau have a different structural sugar metabolic response to WSS. The proteomic data indicate that Scholar may activate the UDP-glucose pathway to produce structural sugars to provide further mechanical support upon WSS infestation; however, the expected decrease in the amount of xylose and arabitol sugars was not observed, suggesting that these sugars are rapidly consumed for secondary cell wall formation. Another method of synthesizing UDP-glucose and supporting the gluconic acid pathway is to oxidize myo-inositol. Several studies have suggested that myo-inositol-derived UDP-glucose production might be specifically induced in plant cells under sugar starvation to maintain metabolic homeostasis (Valluru and Van den Ende 2011). In Scholar but not in Choteau, a significant increase in myo-inositol abundance was observed following WSS infestation, suggesting that myo-inositol is involved in sugar metabolism in this cultivar. Since Choteau was already more resistant to WSS infestation because of its pith-filled stem structure, its WSS response does not appear to involve activation of the cell wall-associated mechanical tolerance pathways to the same extent as does Scholar. The upregulation of fructokinase-2 in Scholar stems further supports this observation, since this enzyme has important roles in vascular development and secondary cell wall formation (Stein et al. 2016). Instead of structural sugars, Choteau might instead activate sugar metabolism to produce more energy, which could explain its significant increase in sucrose-6-phosphate.

### Putative model of the WSS response in hexaploid wheat

**Fig. 6:**
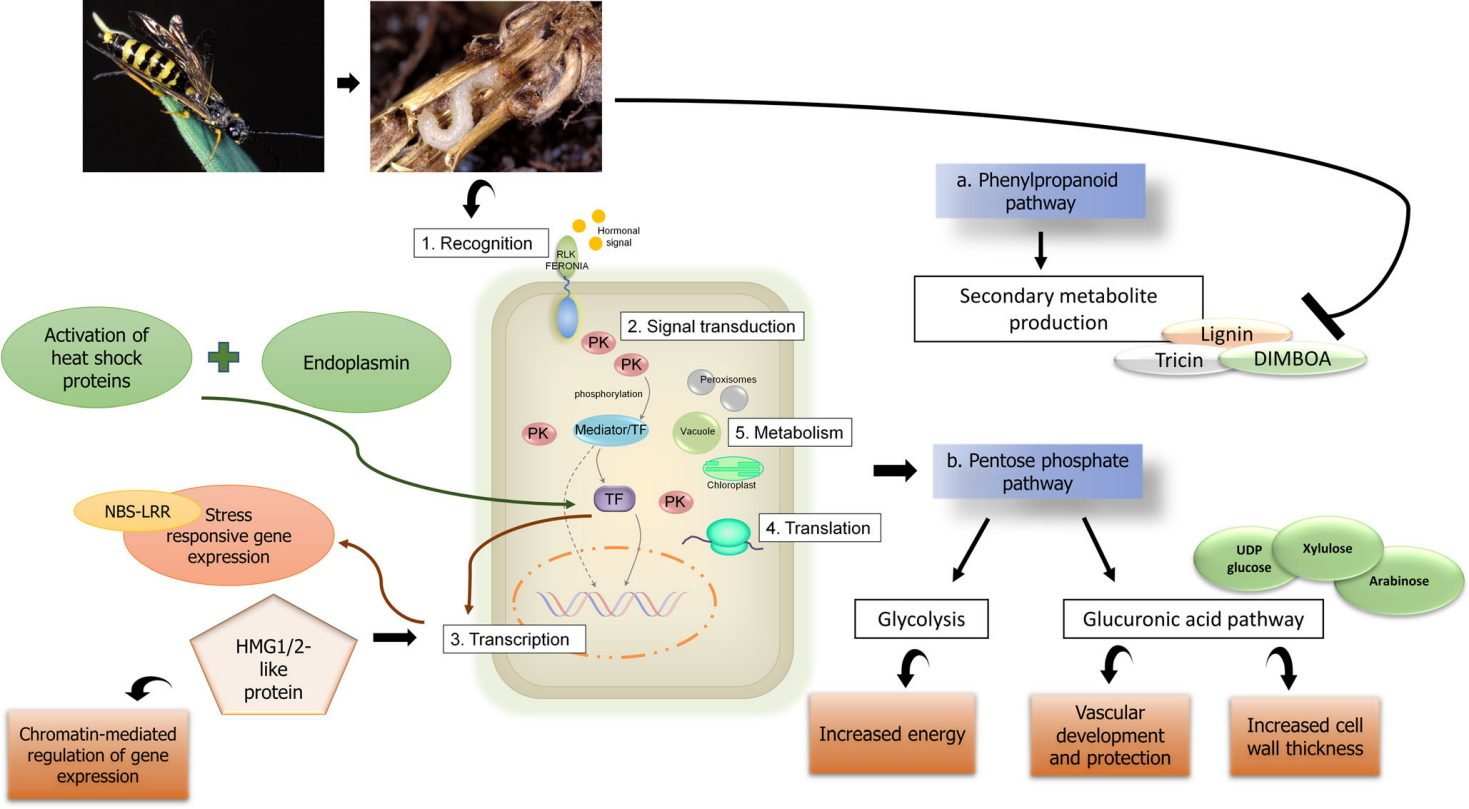
*A putative model for the WSS response in hexaploid wheat.* PK, protein kinase; RLK, receptor-like kinase; TF, transcription factor

Our multiomics approach suggested a putative model for the WSS response in hexaploid wheat in which stem solidness is a key regulator (Fig. 6).

In the putative model of the WSS response, plant hormones (possibly cytokinin) activate a specific response through receptors located in the cell membrane. The activation of heat shock proteins and endoplasmin may also further support this recognition process. The signal is further relayed through the action of a variety of protein kinases, which specifically activate or deactivate mediator proteins through phosphorylation. These mediators then trigger the translocation of specific transcription factors into the nucleus to regulate gene expression. In addition to cellular signaling, chromosomal remodeling may also be involved in the activation of stress-responsive gene expression. This response results in changes to the metabolic events that occur in subcellular compartments, such as the peroxisomes, vacuoles, and chloroplasts. Based on our observations from the multiomics data, it is possible that WSS larvae may target this sequence of events to their benefit. Indeed, oviposition effectors are capable of modulating plant systems, including plant hormones, to suppress the host immune response (Erb et al. 2012). Several studies have provided evidence of herbivorous insects secreting proteins in their saliva that enter plant cells and inhibit the defense signaling pathways (Hogenhout and Bos 2011; Win et al. 2012). The restriction of larval growth by the solid stems of Choteau may result in them being less exposed to the larval effectors, or this cultivar may maintain an active basal level of signaling that can be augmented upon recognition of the larvae, providing better resistance against the developing WSS larvae.

In our model, two metabolic pathways are responsible for the response to WSS: the phenylpropanoid and pentose sugar pathways. The phenylpropanoid pathway produces secondary metabolites that support the mechanical defense against WSS, while also being involved in the production of antibiotic-like compounds that restrict larval growth. The pentose-sugar pathway produces both energy, to support survival under stress conditions, and structural sugar compounds, to provide further mechanical support. Differences in the stem solidness trait may be linked to changes in both of these pathways, since more mechanical support is needed by hollow and semi-solid varieties. Interference with the phenylpropanoid pathway may inhibit the production of compounds such as DIMBOA and tricin, which have an anti-feeding effect on larval growth and oviposition.

Thus, even though stem solidness is an important trait determining the mechanical tolerance to WSS in wheat, the plant’s response to this pest is more complex than simple mechanical restriction of larval growth. We are currently analyzing the functions of some of the identified genes using TILLING and CRISPR mutants. Expanding our understanding of the WSS response mechanisms should further elucidate the interaction between wheat and WSS, facilitating the development of more effective pest control strategies.

## Electronic supplementary material

Supplementary Table S1 Colinearity of the wheat Qss-3BL QTL with corresponding regions in Brachypodium, rice, and barley. (XLSX 65 kb)

Supplementary Table S2 (a) Genome-wide coding targets for putative miRNAs from the *T*. *aestivum* WSS response loci. (b) Genome-wide coding targets for putative miRNAs from the *T*. *turgidum* durum WSS response loci. (c) Genome-wide coding targets for putative miRNAs from the *T*. *turgidum* dicoccoides WSS response loci. (XLSX 20 kb)

Supplementary Table S3 Differentially expressed transcripts between two hexaploid wheat genotypes with different stem solidness, with and without WSS infestation. (XLSX 540 kb)
